# Local Anesthesia and Obtundents

**Published:** 1896-06

**Authors:** 


ARTICLE VII.

### LOCAL ANESTHESIA AND OBTUNDENTS.

(Discussion following the reading of a paper by C. H. Nicholson, D. D. S., before the Rochester Dental Society, Rochester, N. Y.)

Dr. F. J. Woodworth thought one of the principal causes of unsatisfactory results from the use of local anesthetics was the deterioration of the compound. He therefore favored Dr. Hoff's method of reducing the preparation to convenient tablet form, making the solution fresh as needed.

Cleanliness, as nearly absolute as possible, could not be too strenuously insisted upon. Ill after-effects were due oftener probably to half-cleansed instruments than to the medicament, though that, as already suggested, did sometimes deteriorate, when other than bad results were not to be looked for.

He also pronounced strongly in favor of having antidotes, instruments and appliances at hand in case of untoward happenings. Too often these are far removed, thus placing the operator at a disadvantage and making of him, to some extent, a spectacle.

Hemorrhage of the most liberal kind was to be effected. This rid the parts of much of the material injected and reduced to a minimum the chances of inflammation and sloughing.

He preferred, as a generic term, animal magnetism, making two states, the psychologic and the hypnotic. He thought the former, if developed and practiced by the dentist, could be made to answer dental purposes much better than the latter, in that in the hypnotic state the patient loses consciousness and personal control, which necessitate the difficult operation of lodging the suggestion; whereas in the psychologic state the patient retains consciousness and individual control, but is influenced by every mental suggestion prescribed. Hypnotic treatment was objectionable because of the lost time it entailed, while the psychologic treatment was effective forthwith, and directly as the operator acquired the confidence and undivided attention of the patient.

Dr. L. H. Gilbert had had but little experience with general or local anesthetics. The prejudice on the part of the general public was perhaps well founded, but this could be removed in time by proper means, also the unquestioned character of the men by whom it might be practiced.

Dr. B. S. Hert thought obtundents necessitated often by the use of unfit instruments. Good instruments, properly formed and keen-edged, should be used, with or without obtundents, and the results would prove in many cases even more satisfactory to both patient and operator than the average instrument of the average dentist with an obtundent. Those agents are abused in that they are called upon to do what could be better done by suitable instruments in their best possible condition.

He asked Dr. Woodworth to distinguish again between psychologic and hypnotic states; to which the doctor replied: "In the former we have consciousness and voluntary motion, in the latter unconsciousness and immobility."

Dr. F. A. Greene, Geneva, said to get the greatest benefit of an anesthetic the dentist himself must have full faith in it. Any failure in this on his part was fatal to the best results.

Dr. P. H. Smith said the needle had superseded the gas outfit almost wholly in his practice. He had used less than a cylinder of nitrous oxide in a year, extracting and all other operations included.

Dr. F. H. Rood commented on the essayist's statement that he had effected anesthesia with half-strength listerine, and ventured the thought that he had supplied the other half hypnotically, perhaps unconsciously.

Dr. F. H. Sibley used local anesthetics, always subjecting the parts so affected to liberal hemorrhage.

Attention was called to the fact by a member whose name escaped the reporter, that Dr. Lewis, of St. Louis, had found pressure an important factor in the production of local anesthetic effects. Anemia also played a part in the success of the operation. The same had been found true of temperature. Again, density of the fluid had to be considered; the character and strength of the drugs; the temperature of the drugs; the condition of the tissues; the maintenance of edema. Sugar, salt, bromide of potassium, morphine, carbolic acid, caffeine, etc., had been used with more or less satisfactory results. There seemed to be a constant relation between the quantity of a given salt and the complete anesthetic effect.

Dr. W. A. White.—Phelps said he was a country dentist, therefore not a specialist, and while he believed in and used local anesthetics, he always let his dental friends do the experimenting, he taking their summary of results for what he happened to think them worth. He made no promises to the patient beyond what other patients had told him.

As an obtundent fcr sensitive dentine he loudly praised carbolized resin. One of his most sensitive teeth had been operated upon by Dr. Greene, after treatment with this preparation, and the result was such thas he had made frequent use of it ever since. Dr. Greene had not been without it for fifteen years. It had been on the market all this time; but for some reason was not fully appreciated. It should be applied twenty-four hours before excavating the cavity.

He criticised some of Dr. Woodworth's statements in regard to hypnotics. The subjects were not asleep; on the contrary, they were conscious of what was going on, but not necessarily sensible to physical impressions. They were conscious of pins being thrust through the hand, but utterly oblivious to pain. His own experience as a subject, familiar to every one present, left no doubt as to the efficacy of hopnotic treatment.

Dr. I. F. Edington thought dentists had a large field in which to work. The first essential in hypnotic treatment was the fixing of the mind on a given thing. Dentists are poor subjects, as a rule, difficult to control; it is their habit to control others. This was given by way of explanation of the poor results attained at a special meeting of the society, by a professional hypnotist, and who failed utterly.

In closing the discussion, Dr. Nicholson held that the dentist should provide himself with the best of instruments. They were too inexpensive to be omitted by any one, no matter how impecunious.

As to pressure or mere mechanical effect of the injection, there could be no question. Listerine would do the work, pure or diluted. Cocaine should have time, but less than is usually given. He extracted immediately when he used cocaine. He coupled with the drug effect a mental impression, and sent it home by lancing, telling the patient that he was about to lance, that he was lancing, that he had lanced. This was effective in that it was painless and the patient knew it. This prepared the patient for the painlessness, or belief in it, which was practically the same thing, of the operation to follow.

As to the charge of indulgence in "tricks," brought against the professional hypnotists, they were doing only what every successful dentist daily resorted to. We all had tricks—legitimate ones, too—the successful playing of which was to some extent essential to successful practice. Trickery of the legitimate kind is no more against the professional hypnotist than their own little doings were against the dentist. He held that dentists should familiarize themselves with the subject and get out all there was in it.—*The Odontographic.*

